# Structural and biochemical characterization of the essential DsbA-like disulfide bond forming protein from *Mycobacterium tuberculosis*

**DOI:** 10.1186/1472-6807-13-23

**Published:** 2013-10-18

**Authors:** Nicholas Chim, Christine A Harmston, David J Guzman, Celia W Goulding

**Affiliations:** 1 Departments of Molecular Biology and Biochemistry, UCI, Irvine, CA 92697, USA; 2 Pharmaceutical Sciences, UCI, Irvine, CA 92697, USA

**Keywords:** *Mycobacterium tuberculosis*, Disulfide bond, X-ray crystallography, DsbA, Vitamin K epoxide reductase, Oxidoreductase

## Abstract

**Background:**

Bacterial *D*i*s*ulfide *b*ond forming (Dsb) proteins facilitate proper folding and disulfide bond formation of periplasmic and secreted proteins. Previously, we have shown that *Mycobacterium tuberculosis* Mt-DsbE and Mt-DsbF aid *in vitro* oxidative folding of proteins. The *M. tuberculosis* proteome contains another predicted membrane-tethered Dsb protein, Mt-DsbA, which is encoded by an essential gene.

**Results:**

Herein, we present structural and biochemical analyses of Mt-DsbA. The X-ray crystal structure of Mt-DsbA reveals a two-domain structure, comprising a canonical thioredoxin domain with the conserved CXXC active site cysteines in their reduced form, and an inserted α-helical domain containing a structural disulfide bond. The overall fold of Mt-DsbA resembles that of other DsbA-like proteins and not Mt-DsbE or Mt-DsbF. Biochemical characterization demonstrates that, unlike Mt-DsbE and Mt-DsbF, Mt-DsbA is unable to oxidatively fold reduced, denatured hirudin. Moreover, on the substrates tested in this study, Mt-DsbA has disulfide bond isomerase activity contrary to Mt-DsbE and Mt-DsbF.

**Conclusion:**

These results suggest that Mt-DsbA acts upon a distinct subset of substrates as compared to Mt-DsbE and Mt-DsbF. One could speculate that Mt-DsbE and Mt-DsbF are functionally redundant whereas Mt-DsbA is not, offering an explanation for the essentiality of Mt-DsbA in *M. tuberculosis.*

## Background

Correct folding and disulfide bond formation is essential for the function of many secreted proteins including bacterial toxins, and their formation is facilitated by *d*i*s*ulfide *b*ond forming (Dsb) oxidoreductase proteins, which usually contain a conserved thioredoxin (TRX) fold [1]. Protein disulfide bonds can serve structural roles, and thus are often buried in the core of a protein. However, in the case of Dsb proteins, partially exposed disulfide bonds in the TRX-fold CXXC motif have catalytic roles in protein folding, electron transport and bioenergetics in a variety of organisms [2,3].

The Dsb proteins of *Escherichia coli* are the best characterized, and reside in its periplasm to correctly fold disulfide bond containing secreted and cell-wall proteins [4]. *E. coli* DsbA (Ec-DsbA) catalyzes the oxidation of disulfide bonds in reduced, unfolded proteins [5,6], and is then re-oxidized by ubiquinone *via E. coli* DsbB (Ec-DsbB), an inner membrane transmembrane protein, which in turn is oxidized by the electron transport pathway [7,8]. *E. coli* DsbC (Ec-DsbC) and *E. coli* DsbG (Ec-DsbG) serve as proofreading disulfide isomerases that are able to break and correctly reform non-native protein disulfide bonds, thus ensuring that these important secreted proteins are functionally active [9,10]. *E. coli* DsbD (Ec-DsbD) is responsible for maintaining Ec-DsbC and Ec-DsbG in their active redox states and is a transmembrane protein also spanning the inner membrane [11]. Finally, *E. coli* DsbE (Ec-DsbE) is a reductant involved in cytochrome *c* maturation [12], its redox partner is also proposed to be Ec-DsbD [13]. Dsb-like homologs have been found in many prokaryotes [2], including gram-positive bacteria where they also appear to be widespread. As gram-positive bacteria have no spatially defined periplasmic compartment, the precise function and membrane bound redox partners of these Dsb-like proteins appear to differ from those of gram-negative bacteria [14].

Dsb proteins, and in particularly DsbA, have been shown to be involved in virulence of toxin-secreting gram-negative bacteria such as *Yersinia pestis *[15], *Shigella* sp. [16], *Vibrio cholerae *[17,18] and *E. coli *[19]. *Mycobacterium tuberculosis* (Mtb) is a pathogenic bacterium responsible for tuberculosis (TB), which causes approximately 1.4 million deaths and 8 million new cases per year [20]. Mtb secreted proteins have many different functions including those associated with virulence, pathogenicity and cell-wall maintenance. Within the Mtb proteome, it has been predicted that over 180 proteins are secreted, of which ~ 60% may contain disulfide bonds based on their cysteine content, thus suggesting that Dsb proteins may play an important role in the correct folding of secreted proteins [21]. Mtb EspA is one such secreted protein that may require the folding assistance of Mtb Dsb proteins. The single disulfide bond within Mtb EspA has been found to have an important role in disease progression in mice as well as maintaining cell wall integrity [22], highlighting a crucial link between disulfide bond formation and virulence in Mtb. One could speculate that interruption of the Mtb Dsb-assisted folding pathways may prevent mycobacterial infectivity and viability. Therefore, the study of Mtb Dsb protein systems may offer new insight into its virulence and may provide novel anti-TB drug targets.

**Figure 1:**
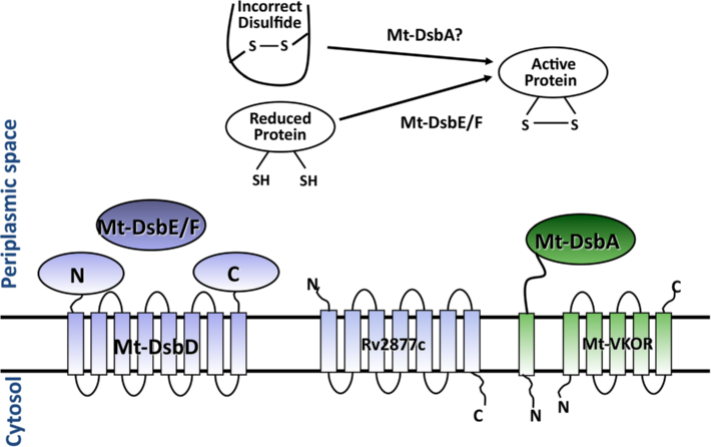
**Two parallel oxidoreductase systems are depicted for disulfide bond formation of Mtb secreted proteins.** Mt-DsbD (Rv2874) and/or Rv2877c are predicted to maintain the redox states of Mt-DsbE (Rv2878c) and Mt-DsbF (Rv1677) while Mt-VKOR (Rv2968c) is proposed to maintain the redox state of its genomic neighbor, Mt-DsbA (Rv2969c).

In Mtb, there are thought to be two distinct Dsb systems, shown in Figure 1. The first system is proposed to have two periplasmic proteins, Mtb DsbE (Mt-DsbE, Rv2878c) and DsbF (Mt-DsbF, Rv1677), and their inner membrane redox partner is thought to be Mtb DsbD (Mt-DsbD, Rv2874) [21] or Rv2877c [23]. Unlike the reductant Ec-DsbE [24], both Mt-DsbE and Mt-DsbF are oxidants as confirmed by their ability to oxidatively fold hirudin [21,25]. The second proposed oxidoreductase system is the Mtb Rv2969c/Rv2968c system [26-28]; Rv2968c is annotated as a vitamin K epoxide reductase (Mt-VKOR) and Rv2969c as a hypothetical protein [29]. As Rv2969c has high sequence identity to gram-positive bacterial DsbA homologs, *Bacillus subtilis* BdbD (Bs-BdbD) and *Staphylococcus aureus* DsbA (Sa-DsbA) [30,31], we shall refer to Rv2969c as Mt-DsbA hereafter. A recent report suggests that Mt-DsbA both localizes on the mycobacterial surface and is associated with the membrane [32], and thus is probably membrane-tethered. Since *Rv2968c* and *Rv2969c* are within the same operon and because Mt-VKOR can functionally substitute Ec-DsbB *in vivo*[27,28], Mt-VKOR and Mt-DsbA may function together [33]. Interestingly, an exhaustive transposon mutagenesis study showed that the genes encoding Mt-VKOR and Mt-DsbA are essential for optimal Mtb growth, whereas Mt-DsbE and Mt-DsbF are not [34], implying that Mt-DsbA is an essential Mtb protein.

In this study, we have determined the 1.9 Å-resolution structure of the soluble form of Mt-DsbA (residues 46–255). The overall fold of Mt-DsbA is reminiscent of Ec-DsbA [32] and consists of two domains, a TRX domain and an inserted α-helical domain. The Mt-DsbA TRX domain active site CXXC motif cysteines are reduced while a stabilizing disulfide bond is observed in the α-helical domain [35,36]. Unlike Mt-DsbE and Mt-DsbF, Mt-DsbA does not have the ability to catalyze the oxidative folding of hirudin [21,25]. However Mt-DsbA possesses disulfide bond isomerase activity as confirmed by its ability to catalyze the refolding of scrambled ribonuclease A (scRNaseA) whereas Mt-DsbE and Mt-DsbF do not. This study represents the structural and functional characterization of an essential Mtb Dsb protein, Mt-DsbA, and suggests that Mt-DsbA likely acts on a distinct protein substrate set as compared to Mt-DsbE and Mt-DsbF due to their functional differences.

## Results

### The structure of Mt-DsbA

Mt-DsbA is predicted to be either secreted into the periplasm by a signal peptide (SignalP) [37] or tethered to the inner membrane by an N-terminal transmembrane helix (TMHMM) [38]. A recent paper suggested that Mt-DsbA is a membrane-tethered protein [32], prompting the investigation of the soluble form of Mt-DsbA encoding residues 46–255, as suggested by TMHMM.

**Figure 2:**
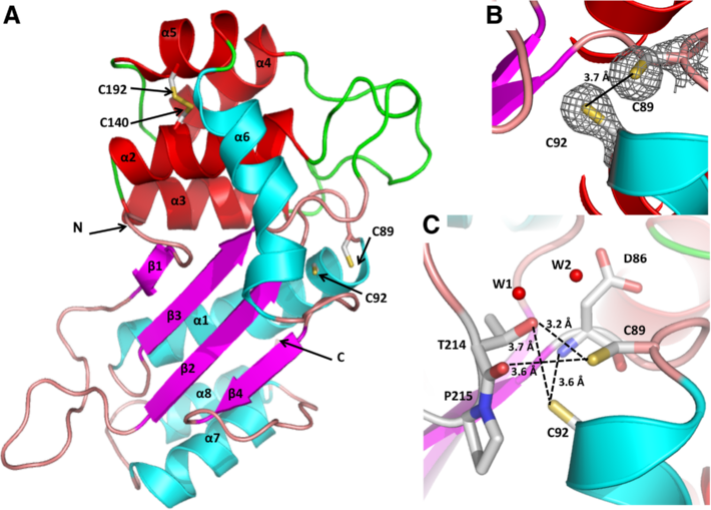
**Crystal structure of Mt-DsbA reveals a DsbA-like fold. A.** Cartoon representation of Mt-DsbA reveals a two-domain structure comprising a TRX domain (colored in pink for β-strands, cyan for α-helices and salmon for loops) and an inserted α-helical domain (colored in red for α-helices and green for loops). The overall fold is reminiscent of DsbA-like homologs. The CXXC motif cysteines, Cys89 and Cys92, are reduced, while the α-helical domain cysteines, Cys140 and Cys192, form a disulfide bond. **B.***2Fo*-*Fc* electron density mesh (grey) of the active site CXXC cysteine residues contoured at 1σ. **C.** The reduced CXXC form of Mt-DsbA is stabilized by hydrogen bonds (designated with black dashed lines) to Thr214 of the *cis*-Pro loop.

The crystal structure of the soluble form of Mt-DsbA was solved by molecular replacement utilizing the model of Bs-BdbD (PDB ID: 3EU3 [39]), which shares 20% sequence identity with Mt-DsbA, combined with the location of two selenium atoms determined from the anomalous data. The structure of Mt-DsbA was solved to 1.9 Å resolution, with two molecules in the asymmetric unit, and the final model had an R_work_/R_free_ (%) of 20.2/23.8. Within the asymmetric unit, residues 56–255 from monomer A and residues 49–255 from monomer B were built into the electron density map while the remaining N-terminal residues were presumed to be disordered as there was no observable density. Monomers A and B are structurally similar and superimpose with a root-mean-square deviation (rmsd) of 0.5 Å. Each Mt-DsbA monomer comprises two domains (Figure 2A), a TRX domain (residues 56–82 and 129–255) and an inserted α-helical domain (residues 83–128), reminiscent of the overall fold of Ec-DsbA [40]. The TRX domain contains the canonical CXXC motif and consists of a four-stranded mixed β-sheet (β1-4) decorated with four α-helices (α1 and α6-8). The α-helical domain inserted between β3 and α6 of the TRX domain comprises four α-helices (α2-5), and is stabilized by a structural disulfide bond between helices α2 and α5 (Cys140 and Cys192 with a Sγ - Sγ distance of 2.1 Å), despite 100 mM DTT in the crystallization condition. The long helix α6 transitions from the α-helical domain back to the TRX domain. The CXXC active site cysteines, adopt a right-handed hook conformation at the N-terminal of helix α1 where the solvent exposed Cys89 and solvent buried Cys92 are reduced with a distance of 3.7 Å between the two Sγ atoms (Figure 2B). The Mt-DsbA *cis*-Pro loop (Thr214-Pro215-Thr216), a conserved feature within the redox-active TRX family, interacts with the CXXC motif through Thr214, which hydrogen bonds to both Cys89 and Cys92 (Figure 2C). The Sγ atom of Cys92 is stabilized by weak hydrogen bonds to the amide N atom of Asp86 (3.6 Å), the Thr214 hydroxyl Oγ atom (3.7 Å) while the Sy atom of Cys89 is hydrogen bonded to the Thr214 hydroxyl Oγ atom (3.2 Å) and its backbone carbonyl O atom (3.6 Å). Additionally, Cys89 is hydrogen bonded to two water molecules (W1 & W2, Figure 2C), and one of these waters (W1) in turn forms a hydrogen bond with Thr214 hydroxyl Oγ atom and its backbone carbonyl O atom.

### Mt-DsbA has structural similarity to other bacterial DsbA proteins

**Figure 3:**
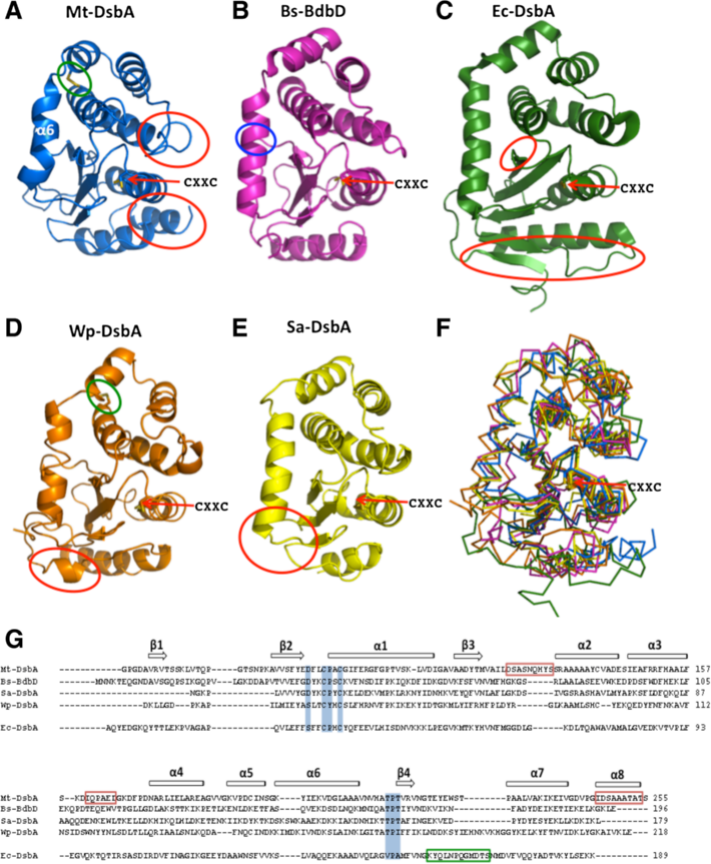
**Structural homologs of Mt-DsbA and sequence alignment.** Structures in panels **A**-**E** are shown in cartoon representation with the CXXC (designated with a red arrow) and structural disulfide bond (designated with a green circle) shown in stick representation with sulfur atoms color in yellow. The blue circle represents the Ca^2+^-binding site of Bs-BdbD and the red circles represent the distinct structural differences between the DsbA-like proteins. **A.** Mtb DsbA (Mt-DsbA, PDB ID: 4IHU) colored in blue **B.***B. subtilis* BdbD **(**Bs-BdbD, PDB ID: 3EU3, [30]) colored in pink **C.***E.* coli DsbA (Ec-DsbA, PDB ID: 1DSB, [40]) colored in green **D.***W. pipientis* α-DsbA (Wp-DsbA, PDB ID: 3F4T, [42]) colored in orange, **E.***S. aureus* DsbA (Sa-DsbA, PDB ID: 3BCI, [31]) colored in yellow **F.** Superimposition of Mt-DsbA with the other four DsbA homologs in ribbon representation. **G.** Overall sequence and structural comparison of Mt-DsbA, Bs-BdbD, Sa-DsbA, Wp-DsbA and Ec-DsbA. The sequences are less than 20% identical and the conserved CXXC and cis-Pro loop motifs are highlighted in blue. Boxed regions correspond to insertions that contribute to structural differences in the respective homolog sequence, red for Mt-DsbA and green for Ec-DsbA. Secondary-structure elements for Mt-DsbA are depicted on the top line.

A structural homology search using the DALI server [41] showed that Mt-DsbA has high structural homology with bacterial DsbA-like proteins (Figure 3F). The two closest structural homologs to Mt-DsbA (Figure 3A) are gram-positive bacteria, Bs-BdbD (Figure 3B) [30] and Sa-DsbA (Figure 3E) [31] with rmsd values of 2.6 Å over 186 Cα atoms and 2.2 Å over 165 Cα atoms, respectively. Of note, Bs-BdbD contains a novel metal binding site at an interdomain position [30], which is not observed in other DsbA homologs (Figure 3B). The next closest structural homolog is a DsbA-like protein from gram-negative bacteria *Wolbachia pipientis* (Wp-DsbA, Figure 3D) [42], which also contains a structural disulfide bond in its inserted α-helical domain as observed for Mt-DsbA [42]. Ec-DsbA also has structural homology with Mt-DsbA with an rmsd of 3.6 Å over 188 Cα atoms (Figure 3C) [43]. There are two main distinct structural features of Mt-DsbA compared to the above DsbA homologs. First Mt-DsbA has an extra C-terminal helix (α8), Figures 2A &3G. Second, within the inserted α-helical domain there are two extended loop regions connecting β3 to α2, and α3 to α4, both within the vicinity of the CXXC active site (Figures 2A &3G). Further, the outer β-strands of the central β-sheet of the TRX domain varies between Ec-DsbA and Mt-DsbA (as well as Bs-BdbD, Sa-DsbA and Wp-DsbA), where Mt-DsbA has an extra strand (β1) at the N-terminal of its β-sheet compared to Ec-DsbA, while Ec-DsbA has two extra β-strands at the C-terminal of its β-sheet compared to Mt-DsbA. Additionally, Ec-DsbA has an extended loop region between its C-terminal β-strand and α-helix, which contributes to the formation of the deep CXXC hydrophobic binding pocket (elaborated upon in the discussion) required for its interaction with Ec-DsbB [44] but not present in Mt-DsbA or the other DsbA homologs (Figure 3).

### Mt-DsbA cannot oxidatively fold reduced and denatured hirudin

**Figure 4:**
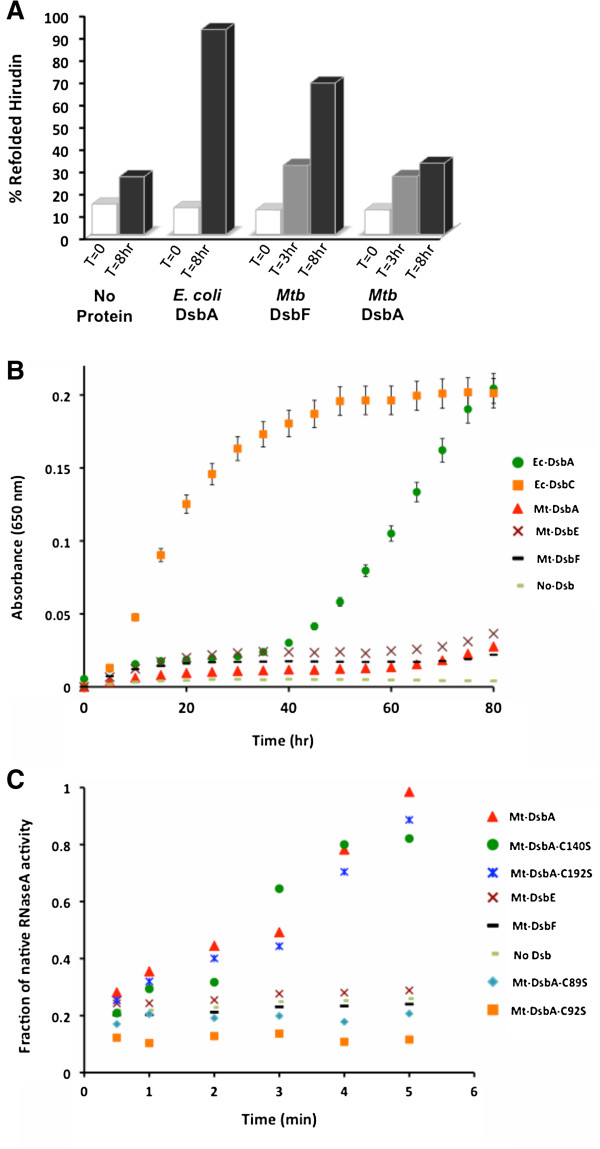
**Biochemical characterization of Mt-DsbA. A.** Mt-DsbA does not facilitate correct disulfide bond formation and folding of denatured hirudin, whereas Mt-DsbF and Ec-DsbA do. **B.** Mt-DsbA does not possess insulin reductase activity as observed for Mt-DsbE and Mt-DsbF. In contrast, Ec-DsbC possesses disulfide reductase activity, and Ec-DsbA has reduced reductase activity in comparison to Ec-DsbC. **C.***In vitro* Dsb protein isomerase activity was assessed by using the scrambled RNaseA (scRNaseA) refolding assay. Mt-DsbA has disulfide bond isomerase activity in contrast to Mt-DsbE and Mt-DsbF, which do not possess the ability to refold scRNaseA. The fraction of native RNaseA activity were calculated and plotted against incubation time.

Since Mt-DsbE and Mt-DsbF have the ability to oxidatively fold reduced and denatured *Hirudo medicinalis* hirudin [21,25], we tested if Mt-DsbA is able to function similarly. The thrombin inhibitor, hirudin, is a 6.9 kDa protein that contains three intramolecular disulfide bonds. MALDI-TOF mass spectrometry analysis of commercial native hirudin revealed significant impurities as well as a major hirudin peak (*m/z* 6765) followed by several smaller hirudin peaks ranging up to *m/z* 7088, consistent with previous reports that hirudin is a non-homogenous protein that contains several variants [45]. Reverse-phase HPLC was used to enhance the homogeneity of the *m/z* 6765 peak as well as remove most contaminating proteins. MALDI-TOF mass spectrometry of HPLC-purified reduced, unfolded hirudin showed a uniform *m/z* increase of 6 Da. Furthermore, the addition of iodoacetamide to reduced, unfolded hirudin carbamidomethylated the free cysteines and resulted in a mass increase of six 57-Da increments (+ 342 Da mass). Thus, the major MALDI-TOF peak was used to monitor the regeneration of reduced, unfolded hirudin (~1.5 pmol) to the fully oxidized native state in the absence and presence of greater than three molar equivalents of Mt-DsbA, Mt-DsbF or Ec-DsbA. At various time points, samples from the folding assay were quenched by the addition of iodoacetamide and analyzed by MALDI-TOF mass spectrometry. The appearance of native hirudin (*m/z* 6765) is represented as a percentage of the total intensities of native and carbamidomethylated hirudin. In the absence of Dsb protein, a small fraction of native hirudin was observed after 8 hours (Figure 4A), presumably due to spontaneous, air-mediated oxidation [21]. As previously reported, Mt-DsbF is able to reoxidize hirudin, and after 8 hours of incubation with Mt-DsbF, approximately 70% of hirudin was in its native state compared to 100% when incubated with Ec-DsbA [25]. In contrast, after an 8-hour incubation with Mt-DsbA, a small fraction of native hirudin equivalent to that of the spontaneous oxidative folding of hirudin in the absence of Dsb protein was observed (Figure 4A).

### Mt-DsbA does not have the ability to reduce insulin

Insulin contains two polypeptide chains (A and B) and has one intramolecular and two intermolecular disulfide bonds. Reduction of these disulfide bonds results in the dissociation of chains A and B, where chain B is insoluble and aggregates. Thus, the reduction of insulin may be assessed by following the increase in turbidity at 650 nm, which is due to the aggregation of chain B and can be determined in the presence and absence of a disulfide reductase. We determined the rate of insulin reduction in the presence of either Mt-DsbA, Mt-DsbE, Mt-DsbF or positive controls (Ec-DsbA and Ec-DsbC). Ec-DsbC and Ec-DsbA both possess insulin reductase activity; however Ec-DsbC is a stronger reductase than Ec-DsbA (Figure 4B). In contrast, in the presence of Mt-DsbA, Mt-DsbE and Mt-DsbF insulin exhibited basal-levels of aggregation similar to that of insulin in the absence of Dsb protein, suggesting that these Dsb proteins are unable to reduce insulin under the conditions tested (Figure 4B).

### Mt-DsbA is able to refold scrambled RNaseA

Mt-DsbA contains some residues characteristic of the *E. coli* disulfide bond isomerases Ec-DsbC and Ec-DsbG (Asp in the DXXCXYC motif, Thr in *cis*-Pro loop). To determine whether Mt-DsbA has isomerase activity, we tested Mt-DsbA catalyzed recovery of active RNaseA from oxidized, disulfide-scrambled RNaseA (scRNaseA). This assay revealed that Mt-DsbA possesses scRNaseA isomerase activity and the active site CXXC cysteines are required for activity, as Cys89Ser and Cys92Ser mutations render Mt-DsbA inactive (Figure 4C). However, the structural disulfide bond in the inserted α-helical domain does not play a role in this activity as the Cys140Ser and Cys192Ser mutants retain similar isomerase activity to wild-type Mt-DsbA (Figure 4C). Of note, Wp-DsbA also contains a structural disulfide bond in its α-helical domain (Figure 3D), and mutation of these cysteines to alanines does not affect its disulfide bond isomerase activity [42], as observed for Mt-DsbA. Moreover, neither Mtb-DsbE nor Mt-DsbF can catalyze the refolding of scRNaseA to produce active RNaseA, and thus do not appear to have isomerase activity under the conditions tested (Figure 4C). These results demonstrate that Mt-DsbA has protein disulfide isomerase activity, while Mt-DsbE and Mt-DsbF do not possess this activity on the substrates tested in this study.

## Discussion

### Comparisons of molecular surfaces and functions of DsbA-like proteins to Mt-DsbA

**Figure 5:**
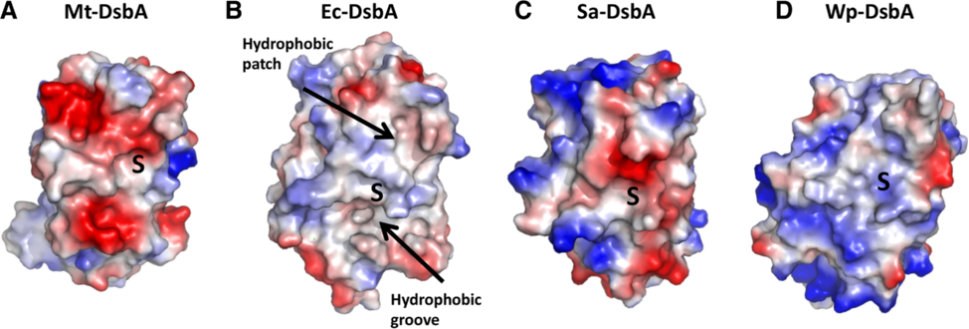
**Electrostatic molecular surface of Mt-DsbA compared to other DsbA homologs. A.** Mt-DsbA (PDB ID: 4IHU) reveals strong negative charges around the hydrophobic active site. **B.** Ec-DsbA, PDB ID: 1DSB, [40]**C.** Sa-DsbA, PDB ID: 3BCI, [31]**D.** Wp-DsbA, PDB ID: 3F4T, [42]. Positive and negative electrostatic potentials, shown in blue (+3.0 kT/e) and red (-3.0 kT/e), respectively. S represents the active sites containing the conserved CXXC motifs.

The electrostatic molecular surface representation of Mt-DsbA (Figure 5A) reveals a shallow hydrophobic pocket at the CXXC active site surrounded by negatively charged patches. The residues that contribute to the negatively charged patch above the CXXC motif are within the α-helical domain, Asp123, Glu165 and Asp169, from the two extended loops as well as Asp203 and Glu200 from α5 helix, whereas residues that contribute to the negative patch below the CXXC motif are from the TRX domain, Glu233 and Glu225, positioned in the loop region connecting β4 to α7. For Ec-DsbA (Figure 5B), the characteristic surface features include a deep hydrophobic groove formed by an extended loop region connecting the C-terminal β-strand and α-helix (Figure 3B), which interacts with a periplasmic loop of its redox partner, Ec-DsbB [46]. Furthermore, an additional hydrophobic patch above the CXXC is thought to be important for binding unfolded protein substrates [44,47]. Similar surface charge and architecture is seen in other gram-negative DsbA proteins such as *V. cholerae* TcpG [17] and *Neisseria meningitidis* NmDsbA3 [48], as compared to Ec-DsbA. The gram-positive bacterial Sa-DsbA (Figure 5C) also has a shallow hydrophobic pocket at the CXXC motif as well as a negative patch directly above its CXXC motif; however this negative patch is not as extensive as the one observed for Mt-DsbA (Figure 5A). Of note, Wp-DsbA has a basic electrostatic surface and deep binding groove near its CXXC motif (Figure 5D).

**Figure 6:**
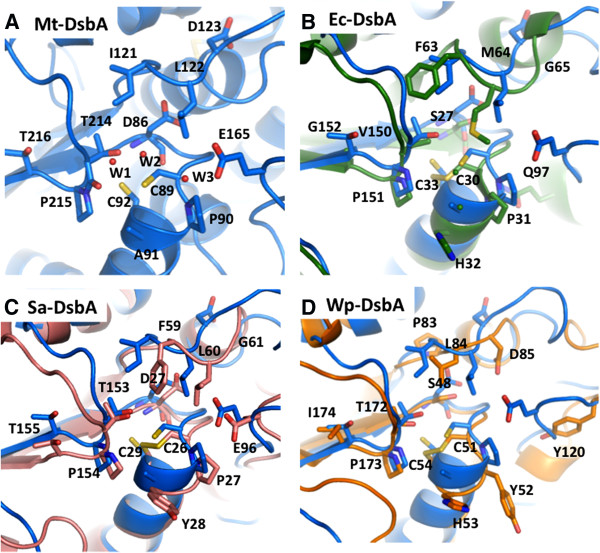
**Comparison of the active sites of DsbA-like proteins.** Proteins are in cartoon representation with notable residues in stick representation, with oxygen, nitrogen and sulfur colored red, blue and yellow, respectively. In panels B-D, active site waters are not shown for clarity. **A.** Mt-DsbA (PDB ID: 4IHU) in blue and waters are depicted as red spheres. **B.** Ec-DsbA (PDB ID: 1DSB, [40]) in green superimposed upon Mt-DsbA in blue, residue numbering for Ec-DsbA. **C.** Sa-DsbA (PDB ID: 3BCI, [31]) in salmon superimposed upon Mt-DsbA in blue, residue numbering for Sa-DsbA. **D.** Wp-DsbA (PDB ID: 3F4T, [42]) in orange superimposed upon Mt-DsbA in blue, residue numbering for Wp-DsbA.

The residues surrounding the CXXC active site motifs are similar among the DsbA-like proteins, however there are subtle differences that may modulate substrate recognition and reactivity. Within Mt-DsbA, Sa-DsbA and Wp-DsbA (Figures 6A, C & D), the residue preceding *cis*-Pro is a Thr, whereas the corresponding Ec-DsbA residue is Val (Figure 6B). In Mt-DsbA, the hydrogen bond between the amide proton of Asp86 and thiolate of Cys89 is also observed in Sa-DsbA (Figure 6C), whereas Asp is substituted with a Ser residue at this position in both Ec-DsbA and Wp-DsbA (Figures 6B & D). The loop region above the active site contains two hydrophobic residues followed by Asp123 in Mt-DsbA (Figure 6A), also observed for Wp-DsbA (Figure 6D), whereas for both Ec-DsbA and Sa-DsbA the charged Asp residue is substituted with Gly (Figures 6B & C). Finally, the third water molecule (W3) observed within the active site vicinity of Mt-DsbA is hydrogen bonded to another water (W2) and also to Glu165, which contributes to the negative molecular surface surrounding the CXXC motif (Figure 6A). A positionally equivalent glutamate residue near the CXXC motif is also observed within the structure of Sa-DsbA (Figure 6C) whereas in Ec-DsbA, Glu165 is substituted by Gln that is not surface-exposed (Figure 6B) and the corresponding residue is Tyr120 in Wp-DsbA, which is considerably further away from the active site as compared to Glu165 of Mt-DsbA (Figure 6D).

Ec-DsbA can catalyze the reduction of insulin in the presence of DTT [42]; in contrast, Mt-DsbA does not show such activity, which may be a consequence of its less hydrophobic patch above the CXXC active site motif (Figures 5A & B). Previous studies of Sa-DsbA demonstrate that, like Mt-DsbA, it has no ability to catalyze the reduction of insulin [31], and both Mt-DsbA and Sa-DsbA have a similar negatively charged molecular surface above the CXXC motif (Figures 5A & C). However, when the preceding *cis-*Pro Sa-DsbA residue, Thr153 was mutated to Val as observed in the Ec-DsbA *cis*-Pro loop (Figures 3G, 6B & C), some insulin reduction activity was restored while Sa-DsbA isomerase activity remained unchanged. In the Wp-DsbA study [42], a similar Thr to Val mutation in the *cis*-Pro motif (Figure 6D) also demonstrated more insulin reductase activity together with reduced isomerase activity compared to wild-type Wp-DsbA, and the Wp-DsbA Thr172Val mutant has a similar activity profile as Ec-DsbA [42]. These observations suggest that the residue preceding *cis*-Pro influences substrate recognition and, in part the activities of Dsb proteins, leading to the speculation that lack of insulin reduction activity for Mt-DsbA may also result from disruption of substrate binding at the negatively charged patch (Figure 5A).

### Structural and functional comparison of Mt-DsbA to Mt-DsbE and Mt-DsbF

**Figure 7:**
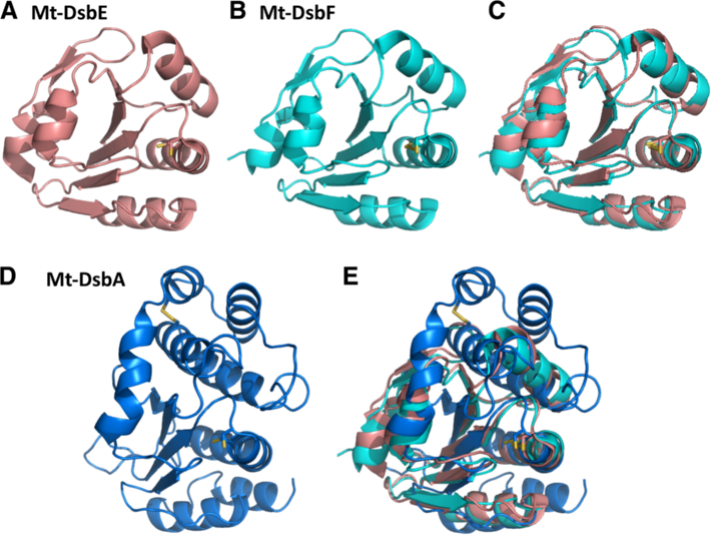
**Mt-DsbA is structurally dissimilar from Mt-DsbE and Mt-DsbF.** Structures in panels A-E are shown in cartoon representation with the CXXC motif and structural cysteines shown in stick representation. **A.** Mt DsbE (PDB ID: 1LU4) colored in light pink, **B.** Mt-DsbF (PDB ID: 3IOS) colored in cyan, **C.** Superimposition of Mtb-DsbE and Mt-DsbF results in an rmsd of 1.0 Å over all Cαs. **D.** Mt-DsbA (PDB ID: 4IHU) colored in blue, **E.** Superimposition of Mt-DsbA with Mtb-DsbE or Mt-DsbF results in the same rmsd of 3.4 Å over all Cαs.

Three secreted or membrane-tethered Mtb Dsb proteins have been structurally and biochemically studied, Mt-DsbE [21], Mt-DsbF [25] and in this study, Mt-DsbA. While Mt-DsbE and Mt-DsbF are structurally similar with an rmsd of 1.0 Å (Figure 7C), Mt-DsbA is structurally distinct (Figures 7D & E). Firstly, Mt-DsbE and Mt-DsbF both consist of one TRX-like domain comprising a five stranded anti-parallel β-sheet decorated by four α-helices (Figures 7A & B) and an additional N-terminal region. In contrast, Mt-DsbA consists of two domains (Figures 2A &7D) and has an overall fold reminiscent of Ec-DsbA [40]. While the core TRX fold for all three Mtb Dsb proteins is conserved, the TRX domains of both Mt-DsbE and Mt-DsbF are more complex compared to that of Mt-DsbA. Both Mt-DsbE and Mt-DsbF have an additional N-terminal region comprising a short 3_10_-helix followed by a β-hairpin and another short 3_10_-helix, and their central β-sheets contain an initial extra β-strand to produce five stranded antiparallel β-sheets compared to the four stranded mixed β-sheet observed for Mt-DsbA. Finally, Mt-DsbA has an extra C-terminal α-helix (α8), which is not present in the structures of Mt-DsbE or Mt-DsbF (Figure 7E). Additionally, the electrostatic molecular surface surrounding the catalytic CXXC motif of Mt-DsbA is more negatively charged than those observed for either Mt-DsbE or Mt-DsbF [25].

Besides structural differences, Mt-DsbA exhibits functional differences compared to Mt-DsbF and Mt-DsbE. Both Mt-DsbE and Mt-DsbF can oxidatively fold reduced and denatured hirudin [21,25] whereas Mt-DsbA does not possess this activity. In contrast, only Mt-DsbA exhibits isomerase/oxidizing activity on the substrate, scRNaseA. These results suggest two features that differentiate Mt-DsbA from Mt-DsbE and Mt-DsbF. First, Mt-DsbA probably functions on a separate subset of Mtb substrates compared to Mt-DsbE and Mt-DsbF. Second, one could postulate that Mt-DsbE and Mt-DsbF have redundant activities even though they have negatively correlated gene expression profiles [25], whereas Mt-DsbA is the sole protein to carry out its unique function in the Mtb Dsb system (Figure 1), and thus *Rv2969c* is an essential gene [34].

**Figure 8:**
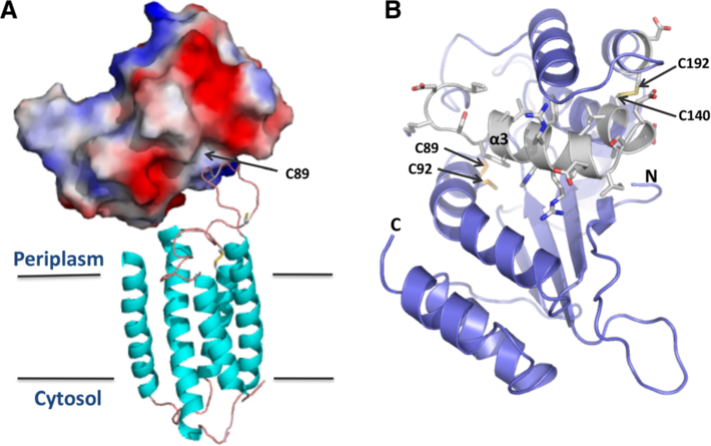
**Mt-DsbA regions proposed to be important for Mt-VKOR interactions and antibody production. A.** Using the *Synechococcus* sp. VKOR/TRX structure (PDB ID: 3KP9) [49], SWISS-MODEL [51] was used to construct a structural homology model of the interaction between Mt-DsbA and Mt-VKOR. **B.** The cartoon representation of Mt-DsbA where white stick representation indicates the 20 amino acid peptide used to produce an antibody that recognized Mt-DsbA on the Mtb cell surface [32].

Since *Rv2968c* and *Rv2969c* are within the same operon and because Mt-VKOR can functionally substitute Ec-DsbB *in vivo*[27,28] and is part of the disulfide bond formation pathway [26], we suggest that membrane-bound Mt-VKOR is responsible for maintaining the redox state of its genomic neighbor, Mt-DsbA. The structure of *Synechococcus* sp. VKOR in complex with its naturally fused thioredoxin-like protein has been solved [49]. We constructed a model of Mt-VKOR based on the *Synechococcus* sp. structure (PDB ID: 3KP9) and modeled Mt-DsbA interacting with the Mt-VKOR loop region which contains two of the four cysteines required for full activity of Mt-VKOR [27], Figure 8A. Interestingly, a previous study has shown that the activity of Mt-VKOR can be inhibited by warfarin, an anticoagulant [27], while another study has shown that small molecules can bind another DsbA-like protein, *V. cholerae* TcpG [50]. As Mt-DsbA and Mt-VKOR are both essential Mtb proteins [34] and are susceptible to small molecules binding, we propose that they may be good candidates for Mtb drug discovery.

### Implications for Mt-DsbA in host-pathogen interactions

A previous study demonstrated that Mt-DsbA localized on the Mtb membrane *in vitro*, and that it also contains several host-cell binding regions [32]. A twenty amino acid Mt-DsbA peptide was used to produce antibodies that demonstrated that Mt-DsbA is exposed on the mycobacterial cell surface. This peptide includes α-helix (α3) within the inserted α-helical region (Figure 8B) and contains many charged residues exposed on the molecular surface of Mt-DsbA. Additionally, it was shown that two other twenty amino acid peptides of Mt-DsbA, one from the N-terminal region (including the first β-strand, β1) and the other from the C-terminal region (including the final α-helix, α8), Figure 2A, have the ability to bind to epithelial cells and inhibit Mtb invasion of these cells in a dose-dependent manner at high micromolar concentrations.

## Conclusions

In this study, we show that Mt-DsbA is structurally distinct from Mt-DsbE and Mt-DsbF. Additionally, unlike Mt-DsbE and Mt-DsbF, Mt-DsbA is unable to oxidatively fold reduced and denatured hirudin but can catalyze the refolding of scRNaseA. Taken together, these results imply that Mt-DsbA functions on a disparate set of substrates compared to either Mt-DsbE or Mt-DsbF. Furthermore, the knowledge that Mt-DsbA possibly facilitates mycobacterial interaction with host cells [32] implicates Mt-DsbA as a potential vaccine candidate. Moreover, as both Mt-DsbA and Mt-VKOR are encoded by essential genes [34], interruption of the Mt-DsbA and Mt-VKOR protein-protein interaction and its redox cycle, may greatly inhibit mycobacterial growth and virulence. Thus further investigation into host protein/Mt-DsbA, small molecule/Mt-DsbA and Mtb protein/Mt-DsbA interactions is warranted, as Mt-DsbA could be an excellent target for novel anti-TB therapeutics.

## Methods

### Cloning and mutagenesis

Mtb *Rv2969c* gene was amplified from Mtb H37Rv genomic DNA using KOD HotStart Polymerase Kit (EMD Millipore) to encode residues 46–255. The 5' primer starts with the *Nde*I restriction site and 3' primer (contains no stop-codon) ends with the *Hind*III restriction site (Table 1). The resulting product was excised from a 2% agarose gel and purified using a gel extraction kit. The PCR product was ligated into a linearized blunt vector, pCR-BluntII-TOPO (Invitrogen), and then transformed into OneShot TOP10 *E. coli* cells (Invitrogen). Presence of the correct gene was confirmed by DNA sequencing (Laguna sequencing, Laguna, CA). *Rv2969c* was double-digested from the blunt vector with *Nde*I and *Hind*III, and the plasmid pET30(+) (EMD Millipore) was digested with the same restriction enzymes. The cut *Rv2969c* gene was then ligated into cut pET30 vector and transformed into *E. coli* BL21(DE3)-Gold cells (Agilent). Presence of the correct gene was confirmed by sequencing (Laguna sequencing, Laguna, CA).

**Table 1:** Primers used to produce the Mt-DsbA construct (residues 46–255)

	**Primers:**
**Forward**	5’ CCCATATGCGCGACGACAAGAAGGACGGCGTCGCGGG 3’
**Reverse**	5’ CCAAGCTTGGATGTCGCGGTAGCAGCGGCCGAGTC 3’

The cysteine to serine mutants were constructed via site-directed mutagenesis. The primers used are listed in Table 2. Presence of the correct mutation was confirmed by sequencing (Laguna sequencing, Laguna, CA).

**Table 2:** Primers used for site-directed mutagenesis to produce Cys to Ser mutants

	**Primers:**
**C89S**	For: 5’ CTTCTACGAGGATTTCCTGTCTCCGGCGTGCGGCATATTC 3’
	Rev: 5’ GAATATGCCGCACGCCGGAGACAGGAAATCCTCGTAGAAG 3’
**C92S**	For: 5’ GAGGATTTCCTGTGTCCGGCGTCCGGCATATTCGAGCGCGGTTCGG 3’
	Rev: 5’ CCGAACCGCGCTCGAATATGCAGGCCGCCGGACACAGGAAATCCTC 3’
**C140S**	For: 5’ CTGCTGCGGCTTATTCCGTTGCCGACGAATC 3’
	Rev: 5’ GATTCGTCGGCAACGGAATAAGCCGCAGCAG 3’
**C192S**	For: 5’ CAAGGTGCCCGACTCCATCAACAGCGGCAAG 3’
	Rev: 5’ CTTGCCGCTGTTGATGGAGTCGGGCACCTTG 3’

### Protein expression and purification

The mature form of Mt-DsbA residues (46–255) with a C-terminal HisTag was overexpressed from a pET30 plasmid containing a truncated *Rv2969c* gene using *E. coli* BL21(DE3)-Gold cells. Cells were grown at 37**°**C in LB medium containing 50 μg/ml of kanamycin. Protein expression was induced by adding 1 mM IPTG at an OD_600_ ~ 0.8 and grown for 4 h before harvesting. Cells were pelleted at 5,000 rpm for 10 min and then resuspended in wash buffer (50 mM Tris–HCl pH 7.4, 350 mM NaCl, 10 mM imidazole and 10% glycerol) containing phenylmethylsulfonyl fluoride and hen egg lysozyme, and then were lysed by sonication and centrifuged at 13,000 rpm for 40 min followed by filtration (0.22 μm) to remove cell debris before purification. The cell lysate was loaded on to a Ni^2+^-charged HiTrap column (5 mL) and washed with wash buffer before protein was eluted with a 10–500 mM linear imidazole gradient (100 ml) in which purified Mt-DsbA eluted between 200 and 300 mM imidazole. The fractions containing pure Mt-DsbA were collected and concentrated (Amicon, 10 kDa molecular mass cutoff) and then further purified by gel filtration on a Superdex 200 column (GE Healthcare) equilibrated with 20 mM Tris–HCl (pH 7.4), 150 mM NaCl using an AKTA FPLC. The selenomethionine-derivatized (SeMet)-Mt-DsbA was grown in M9 minimal medium supplemented with amino acids supplements (leucine, isoleucine, valine, 50 mg/L; phenylalanine, lysine, threonine, 100 mg/L; and selenomethionine 75 mg/L) adapted from a previously described protocol [52]. The SeMet-Mt-DsbA and cysteine to serine mutants were purified as described for native Mt-DsbA.

### Crystallization, data collection and structure determination

Diffraction quality SeMet-Mt-DsbA crystals were grown at room temperature by hanging drop-vapor diffusion with a reservoir containing 2 M ammonium sulfate, and 9% isopropanol and a 75 mg/mL protein solution in 20 mM Tris–HCl (pH 7.4), 150 mM NaCl. The crystals were mounted and diffraction data were collected under cryoconditions by swiping the crystal through a saturated ammonium sulfate solution. X-ray diffraction data of a single SeMet-Mt-DsbA crystal was collected at Beamline 5.0.2 at the Advanced Light Source (Berkeley) to 1.9 Å, using the Se peak wavelength of 0.97602 Å. The data were processed in iMosfilm [53] in P2_1_2_1_2; with unit cell dimensions of 71.0 Å × 76.7 Å × 86.9 Å and two molecules in the asymmetric unit. The resulting mtz file was run through SHELX [54] to determine the location of the Se atoms. The structure of Bs-BdbD, (PDB ID: 3EU3, [39]) was used as a model for molecular replacement in Phaser [55] with the heavy atom coordinates of Se from SHELX [54]. AutoBuild [56] was used to place α-helices and β-strands. The final model of Mt-DsbA was built through iterative manual building in Coot [57] followed by refinement with phenix.refine [43]. The Mt-DsbA final model includes residues 56–255 and 49–255 of chains A and B (respectively), and has a final R_work_/R_free_ (%) of 20.2/23.8. Additionally, Lys168 from monomer A, and Asp160 and Asp242 from B were modeled as alanines as there was no observable electron density for their side-chains. The stereochemistry and geometry of each Mt-DsbA monomer was validated with PROCHECK [58] and ERRAT [59], and was found to be acceptable, except for Val186 from chain A and Ser227 from chain B which despite fitting the electron density well are in the disallowed region. The data collection and refinement statistics are presented in Table 3. All structural figures are generated in PyMOL [46].

**Table 3:** X-ray diffraction data collection and atomic refinement statistics for Mt-DsbA in its reduced form

***Space group***	***P2***_***1***_***2***_***1***_***2***
No of monomers per AS unit	2
Unit cell dimensions (Å)	71.0 × 76.7 × 86.9
pH of crystallization condition	7.4
***Data set***	
Wavelength (Å)	0.976
Resolution range (Å)	44.70-1.90
Unique reflections (total)	38372 (706287)
Completeness (%)^¶^	99.96 (100)
Redundancy	10.7 (10.6)
R_merge_^¶,a^	7.4 (39.3)
I/σ^¶^	5.8 (1.9)
***Model refinement***	
Resolution range (Å)	44.70-1.90
No. of reflections (working/free)	36450/1922
Residues of Mt-DsbA	Chain A 56-255
	Chain B 49-255
No. of protein atoms	5975
No. of water molecules	300
*R*_work_/*R*_free_^b^, %	20.2/23.8
***Ramachandran plot***	
Most favorable region (%)	97.30
Additional allowed region (%)	2.21
Disallowed region (%)	0.49
***PDB ID code***	4IHU

^¶^Statistics for the highest resolution shell are given in (parenthesis).

^a^ R_merge_=Σ|*I*-<*I*>|/Σ*I*.

^b^ R_*work*_=Σ|*F*_obs_-*F*_calc_|/Σ*F*_obs_*R*_free_ was computed similarly for a test set of 5% randomly selected data, which were not used in refinement.

### Oxidation and reduction of Mt-DsbA

To oxidize Mt-DsbA, 50 mM of oxidized glutathione (GSSG) was added to as-isolated Mt-DsbA in 50 mM Tris–HCl, 150 mM NaCl and incubated for 1 hour at room temperature. Oxidized Mt-DsbA was recovered by gel filtration in 50 mM Tris–HCl pH 7.4, 150 mM NaCl. To reduce Mt-DsbA, 100 mM dithiothreitol (DTT) was added to Mt-DsbA and incubated at 4°C overnight. Reduced Mt-DsbA was recovered by gel filtration in 50 mM Tris–HCl, 150 mM NaCl.

The redox state of the thiols was confirmed by the Ellman’s assay, which exploits the colorimetric change at 412 nm when 5,5'-dithiobis-(2-nitrobenzoic acid) (DTNB) is converted to 2-nitro-5-thiobenzoate upon cleavage of the disulfide bond by free thiols. This reaction is stoichiometric, thus allowing for accurate quantification of free thiols.

### To test for Dsb-catalyzed oxidative folding of hirudin

To test for Mt-DsbA catalyzed oxidative folding of reduced, denatured hirudin *in vitro*, the experiment was carried out as previously described [25]. Commercial *H. medicinalis* hirudin (Sigma) was purified to remove contaminants by reverse-phase HPLC on a Synergi 4 mm Hydro-RP column (250 × 4.6 mm, Phenomenex) at a flow rate of 0.5 ml/min. Solvents A and B used for reverse-phase HPLC were 0.1% trifluoroacetic acid in water and acetonitrile, respectively. Purified hirudin was reduced and denatured by incubation with 100 mM DTT, 6 M Gdn-HCl overnight and eventually desalted by ZipTip C4 (Millipore). To test for oxidative folding of reduced, denatured hirudin, 3 molar equivalents of oxidized Mt-DsbA (5 pmol) in 100 mM ammonium bicarbonate, pH 8.0 at 25°C and added to reduced, denatured hirudin (~1.5 pmol). Each 10 μl reaction was quenched at different time points by a 10-minute incubation at 50°C with 50 μl 6 M Gdn-HCl and followed by the addition of 0.5 μl 100 mM iodoacetamide for 15 minutes at 25°C. The samples were then desalted by ZipTip C4 (Millipore) and analyzed by MALDI-TOF mass spectrometry (Voyager). Experiments were repeated with no protein and with approximately three molar equivalents (5 pmol) of Mt-DsbF and Ec-DsbA as positive controls. The appearance of native hirudin (*m/z* 6765) is represented as a percentage of the total intensities of native and carbamidomethylated hirudin [25].

### To test for Dsb-catalyzed reduction of insulin

To test for protein disulfide reductase activity of Mt-DsbA, experiments were carried out *in vitro* using the insulin reduction assay in the presence of DTT [60]. 10 μM of Ec-DsbA, Ec-DsbC (as positive controls, Sigma) or Mt-DsbA in buffer containing 0.1 M phosphate buffer, pH 7.2, 2 mM EDTA and 0.33 mM DTT were mixed with 0.131 mM insulin to initiate the reaction. Insulin comprises A and B polypeptide chains connected by two disulfide bonds. Reduction of the disulfide bonds leads to precipitation of the insoluble B chain, and is followed spectroscopically as an increase in optical density at 650 nm. The solutions were monitored at 30 s intervals over a period of 80 min*.*

### To test for Dsb-catalyzed refolding of scrambled RNaseA

*In vitro* isomerase activity of Dsb proteins were assessed utilizing scRNaseA as previously described [61]. ScRNaseA was produced by first incubating native RNaseA (Sigma) in 50 mM Tris–HCl, pH 8.0 with 6 M Gdn-HCl and 0.1 M DTT overnight at room temperature. The reduced, unfolded RNaseA was acidified with 100 mM acetic acid, pH 4 and purified over a desalting column. The presence of eight free thiols was confirmed by the Ellman’s assay. To generate randomly oxidized disulfide bonds in scRNaseA, reduced RNaseA in 50 mM Tris–HCl pH 8.5 was incubated with 6 M Gdn-HCl in the dark at room temperature for 3 days before being acidified and purified. Oxidization of the disulfide bonds to produce scRNaseA was confirmed by the Ellman’s assay.

Isomerase activity of reduced Mt-DsbA and mutants were tested by measuring spectrophotometrically RNaseA cleavage of cyclic-2’,3’-cytidinemonophosphate (cCMP) to 3’-cytidinemonophosphate (3’CMP), which results in an increase in absorption at 296 nm. Purified Mt-DsbA or mutants (10 μM) were added to 100 mM sodium phosphate pH 7, 1 mM EDTA, and 10 μM DTT at room temperature for 5 minutes. To initiate the reaction, 40 μM scRNaseA was added. At several time points, 20 μl aliquots were taken and added to 60 μl of 4 mM cCMP, so that the final volume was 80 μl. The rate of RNaseA cleavage of cCMP was monitored at 296 nm for 3 min and the percentage of native RNaseA activity was plotted against time.

## Abbreviations

Dsb: Disulfide bond forming proteins; Mtb: *Mycobacterium tuberculosis*; TB: Tuberculosis; PDB: Protein Data Bank; TRX: Thioredoxin; DTT: Dithiothreitol; EDTA: Ethylenediaminetetraacetic acid; IPTG: Isopropyl β-D-1-thiogalactopyranoside; DTNB: 5,5'-dithiobis-(2-nitrobenzoic acid); vitamin K: Epoxide reductase; VKOR: Gdn-HCl, guanidine hydrochloride; MALDI-TOF: Matrix-assisted laser desorption/ionization time-of-flight; HPLC: High-performance liquid chromatography; rmsd: Root-mean-square derivation; RNaseA: Ribonuclease A; scrambled RNaseA: scRNaseA; SAD: Single-wavelength anomalous diffraction; cCMP: Cyclic-2’,3’-cytidinemonophosphate; 3’CMP: 3’-cytidinemonophosphate.

## Competing interests

The authors declared that they have no competing interests.

## Authors’ contributions

NC designed and performed the experiments as well as prepared the manuscript. CAH and DJG performed the experiments. CWG conceived of the study, designed the experiments and prepared the manuscript. All authors read and approved the final manuscript.
